# Decline of semen quality over the last 30 years in Uruguay

**DOI:** 10.1186/s12610-021-00128-6

**Published:** 2021-05-06

**Authors:** Lucía Rosa-Villagrán, Natalibeth Barrera, José Montes, Carlos Riso, Rossana Sapiro

**Affiliations:** 1 Departamento de Histología y Embriología, Facultad de Medicina, Universidad de la República, Gral. Flores 2125, 11800 Montevideo, Uruguay; 2 Laboratorio de Andrología, Fertilab Laboratorio de Análisis Clínicos, Montevideo, Uruguay; 3 Laboratorio de FIV, Centro de Esterilidad Montevideo (CEM), Montevideo, Uruguay

**Keywords:** Male infertility, Sperm donors, Semen analysis, Infertilité masculine, Donneurs de sperme, Analyses de sperme

## Abstract

**Background:**

Over the last years, there has been an increasing concern about a global decline in men’s fertility. Specifically, some evidence indicates that sperm quality has decreased over the last years. However, reports showing the changes in sperm quality with time are inconsistent. Part of the contradictions between studies is attributed to geographical differences. Surprisingly, few studies include data from South American countries, creating a bias in the conclusions. This study aims to determine how sperm quality has evolved over the past 30 years in Uruguay. For this purpose, 317 medical records from allegedly healthy sperm donor candidates, aged between 18 and 36 years old, who voluntarily requested to be considered as sperm donors between 1988 and 2019, were analyzed. The studied variables were the following sperm parameters: semen volume, sperm cell concentration, total sperm number, progressive motility, vitality, and sperm morphology. A correlative statistical analysis was performed between seminal parameter values and the year data were collected.

**Results:**

We found a statistically significant decrease in sperm concentration and normal sperm morphology during the studied period. There was no decrease in vitality, seminal volume, and total progressive motility. Semen parameters were not associated with tobacco, drugs, or alcohol consumption.

**Conclusions:**

We conclude that the sperm quality of donor candidates in Uruguay decreased during this period. Further studies should be carried out to verify the occurrence of this phenomenon in the general population and find its possible causes.

## Résumé

**Contexte:**

Au cours des dernières années, on s’est de plus en plus inquiété d’une baisse de la fertilité masculine. Plus précisément, certaines données indiquent que la qualité du sperme a diminué au cours des dernières années. Cependant, les rapports montrant les changements dans la qualité du sperme avec le temps sont incohérents. Une partie des contradictions entre les études est attribuée à des différences géographiques. Étonnamment, peu d’études ont inclue des données provenant de pays d’Amérique du Sud, ce qui crée un biais dans les conclusions. Cette étude vise à déterminer comment la qualité du sperme a évolué au cours des 30 dernières années en Uruguay. À cette fin, 317 dossiers médicaux de candidats donneurs de sperme prétendument en bonne santé, âgés de 18 à 36 ans, qui ont volontairement demandé à être considérés comme donneurs de sperme entre 1988 et 2019, ont été analysés. Les variables étudiées étaient les paramètres suivants du sperme: volume de sperme, concentration de cellules spermatiques, nombre total de spermatozoïdes, motilité progressive, vitalité, et morphologie des spermatozoïdes. Une analyse statistique corrélative a été effectuée entre les valeurs des paramètres séminaux et l’année de recueil des données.

**Résultats:**

Nous avons trouvé une diminution statistiquement significative de la concentration de spermatozoïdes et de la morphologie normale des spermatozoïdes pendant la période étudiée. Il n’y a eu aucune diminution de la vitalité, du volume de sperme, et de la motilité progressive totale. Les paramètres du sperme n’étaient pas associés à la consommation de tabac, de drogues ou d’alcool.

**Conclusions:**

Nous concluons que la qualité du sperme des candidats donneurs en Uruguay a diminué au cours de cette période. D’autres études devraient être menées pour vérifier l’apparition de ce phénomène dans la population générale et en trouver les causes possibles.

## Background

The total number of couples affected by infertility worldwide has been estimated to be 48.5 million in 2010. The male factor accounts for at least 30–40% of these cases [1]. As a consequence of the increase of the couple’s age at conception, changes in the lifestyle and environmental factors (e.g. increase in pollution) as well as other still unknown factors, it is possible that an even more pronounced increase in the number of infertile couples could be detected, creating, therefore, a global health problem [2–5]. There is an important current controversy regarding the possibility that the male factor -by a decrement of semen quality parameters- may have firmly contributed to the decline of fertility indexes. In 1992, Carlsen et al published a meta-analysis indicating that semen quality had declined annually [6]. Since then, the global trend in semen values has been under constant debate, leading to several controversies. Some reports showed a deterioration of sperm quality [7–10] but others did not [11, 12]. The difference among these findings may be a consequence of changing laboratory methods, statistical issues, heterogeneity of populations selected for studies (fertile or infertile men, geographical regions, ethnic groups) [13]. Moreover semen quality may depend on men’s age and lifestyle factors such as smoking, alcohol consumption, stress, and obesity, among other factors [14–18]. Since part of the variability has been attributed to differences between regions and countries, it is surprising the lack of data including South American countries. The latest data from South America come from very few studies carried out in Brazil [19], Argentina, and Venezuela [20, 21].

In Uruguay, a previous study was performed on 71 sperm donors showing that sperm concentration had decreased in parallel with an increase of semen volume. In general, the study showed that the decrease in sperm concentration had been compensated by an increase in semen volume, keeping the average total sperm number unchanged [22]. However, this study analyzed only donors who had effectively been included in the semen donor program, participating in medically assisted reproduction procedures, meaning that only men with better semen quality, and who had passed strict medical examinations, were included for the study. The fact that only a small population of the group of donor candidates was analyzed may create a bias in the conclusion that sperm quality remained unchanged in the past years.

Considering this perspective, this work aims to analyze if sperm parameter values have decreased during the past years. To achieve the objective, we included all healthy men who applied to be sperm donors, regardless of whether or not they were accepted into the donation program from the same center for the last 30 years. We analyzed not only their spermiograms but also confounding factors such as age, body mass index, tobacco, alcohol, and drug consumption.

## Material and methods

### Study population

We analyzed 386 medical records from allegedly healthy sperm donor candidates, aged between 18 and 36 years old, who voluntarily requested to be considered as sperm donors in a fertility clinic between 1988 and 2019. Of these individuals, 317 had records of their sperm parameter values. Ethical observance of the study was followed as outlined in the declaration of Helsinki and the Institutional Review Board approved the study.

### Inclusion and exclusion criteria

This study included those semen donor candidates who responded to advertising posters and advertisements on the web platform, requesting that they were presumably healthy and were willing to pass a screening test, which included medical history, physical examination, and laboratory tests. To qualify as donors, men complied with three evaluation rounds, starting with an initial interview and physical examination conducted by a physician. During the interview, the potential donor’s family history, lifestyle habits, and motivation for sperm donation were scrutinized. This was followed by a complete semen analysis. Finally, routine blood and urine analyses were performed that included tests to discard metabolic and infectious diseases. Since 2005 a genetic screening for cystic fibrosis has been included.

All candidates were asked to sign an informed consent explaining that their data could be anonymously used for research purposes. Those individuals who refused to give their informed consent were excluded from this study.

### Semen analysis

Semen samples for the consideration of semen parameters were obtained by masturbation after 3 to 5 days of sexual abstinence. The samples were kept unprocessed for about 1 h to allow liquefaction to occur. We used semen parameters of their first ejaculate for statistical analysis. The evaluated parameters in this study were semen volume, sperm concentration, total sperm number, sperm motility, sperm morphology, and sperm vitality. Semen samples were analyzed by manually applied protocols according to the World Health Organization (WHO) guidelines, based on the procedures described in the latest laboratory manual available at the time of sample examinations [23–25].

#### Sperm count

After liquefaction, each sample was transferred to a graduated conical tube with a sterile Pasteur pipette for volume verification. An aliquot was examined by light microscopy using an appropriate counter (Makler chamber). The results of the analyses readings were submitted to a mathematical formula with a correction standard of each dilution to obtain the final concentration of millions of sperm cells per mL (millions/mL) [23–25].

#### Sperm motility

A 10 μL aliquot of the semen sample was placed between two coverslips for double evaluation under light microscopy, following the criteria recommended by WHO manuals. At least 100 spermatozoa were counted in each slide, classifying them as grade a (rapid progressive motility or velocity ≥ 25 μm per second); grade b (slow progressive motility or speed < 25 μm per second): grade c (non-progressive motility or speed < 5 μm per second); or grade d (immobile spermatozoa), showing the percentage results in each category. Motility was also classified as progressive (sum of the percentage of spermatozoa classified as grades a and b) and total motility (sum of the percentage of spermatozoa classified as grades a, b, and c).

#### Sperm morphology

Modified Papanicolaou staining was used to assess sperm morphology. For each sample, two smears were prepared and 200 spermatozoa were assessed. Abnormalities of the sperm head, neck, and tail were evaluated at high magnification (× 1000) using a high-resolution (100×) oil-immersion objective and bright-field microscope optics. The results of sperm morphology staining were expressed as “percentage of abnormal sperm morphology”. Evaluations of sperm morphology were performed using different criteria over the time of the study. Since 1988 and up to December 1999 sperm morphology was analyzed according to WHO manuals available at the time of the exams [23, 25]. Starting the year 2000 Fertilab applied Kruger strict criteria categorization to assess sperm morphology [26].

#### Sperm vitality

Sperm vitality was evaluated by determining the integrity of the membrane of these cells using a dye exclusion method. This method is based on the principle that damaged plasma membranes, as well as those belonging to dead cells, allow the entrance of impermeant stains into the cell. The lower reference limit for vitality (membrane intact sperm) was 58% [25].

### Data collection

For the analysis of the variables mentioned in this study, the necessary data were obtained from the medical records of the Fertilab laboratory database. These medical records were irreversibly dissociated from identification data, thereby ensuring subject anonymity and confidentiality of data. The dissociation of data was carried out by non-research personnel. Record sheets were used for age, weight, height, tobacco, alcohol and drug consumption, semen volume, total sperm number, concentration, motility, morphology, and sperm vitality.

### Statistical analysis

Statistical analysis was performed by using the GraphPad Prism version 8.0.0 for Windows, GraphPad Software, San Diego, California USA, www.graphpad.com”, and the JASP Team (2020), JASP (Version 0.140) statistical software. Data were expressed as median values and interquartile ranges, while means and standard deviations (SD) were also reported. Normal distribution of data was tested with the Shapiro-Wilk normality test. The sperm parameter values were not normally distributed. The correlation of seminal parameters values and the year of sample collection were determined by Spearman correlation test. Linear regression was applied to calculate the modifications of sperm concentration and sperm total number per year.

Either Student’s t-tests or Mann-Whitney test (depending on the distribution of data) were used to compare data between two groups. The comparison between multiple groups was made either by using the one-way ANOVA test followed by Tukey post-test or the Kruskal-Wallis test depending on the distribution of data. Chi-Squared Test was applied to analyze categorical variables. The relation of sperm concentration and sperm motility with the independent variables (age at donation, cigarette consumption, and alcohol abuse) was tested with multiple regression analysis. The best transformation of the data that yielded normal distributions for variables without normal distributions was the logarithmic (base 10) transformation, except for sperm morphology where Johnson transformation was applied. We evaluated the fit of the regression models by testing the residuals for normality and by inspecting the residual plots. BMI data and the number of men who consume drugs were not included in the multiple regression analysis; BMI positively correlated with the age of men while the consumption of drugs was registered only since 2012. The results were considered significant with a two-tailed *p*-value < 0.05.

## Results

### Description of the population

A total of 386 men attended the Andrology Clinic from 1988 to 2019 for sperm donation. A total of 317 men who satisfied the inclusion criteria were included in this study. Their general characteristics are shown in Table 1.

**Table 1:** General characteristics of sperm donors

	Age (years)	Height (cm)	Weight (kg)	BMI (kg/m^**2**^)^a^
N	250	203	195	179
Mean ± SD	24.5 ± 4.3	177.0 ± 7	74.6 ± 8.9	23.7 ± 2.6
Minimum	18	160	55	18.4
Maximum	36	196	105	34.35

Descriptive analysis of individuals included in the study

^a^*BMI* Body mass index, *N* Number of donors, *SD* Standard deviation

Twenty percent of men (42/211) declared that they regularly smoke, 43% of men (91/211) indicated that they regularly consume alcohol, and 24% (24/114) declared regular consumption of other drugs (particularly marijuana). Lacking data in the case of alcohol and tobacco consumption were 33% (116/317) and 66% (210/317) in the case of marijuana.

### Sperm parameter description

Excluding those men who were rejected either because of health problems or because they refused to sign the informed consent to become part of the study, data of 317 men were analyzed. Table 2 summarizes the descriptive statistics of the initial semen evaluation, including volume, sperm concentration, vitality, the percentage of normal sperm morphology, and sperm motility for each man. The mean of all semen parameter values was above the cut-off values recommended by WHO since the year 2010 [25].

**Table 2:** Descriptive characteristics of participants’ semen parameters

	Volume (ml)	Sperm concentration (10^**6**^/ml)	Vitality (%)	Morphology (%)	Motility a (%)	Motility b (%)	a + b (%)
N	317	317	209	313	317	317	316
Mean ± SD	3.5 ± 1.6	64.6 ± 40.9	85.4 ± 12.4	13.8 ± 11.6	28.9. ± 14.4	31.5 ± 11.9	60.2 ± 13.6
Median	3.2	58	90	11	27	32	62
25th centile	2.3	33.9	77.5	8	19	21	52
75th centile	4.5	85.5	95	15.5	38	41	69

The data were obtained by the analysis of the first spermiogram of 317 individuals collected between 1988 and 2019

*N* Number of donors, *SD* Standard deviation

### Semen variations over time

**Fig. 1:**
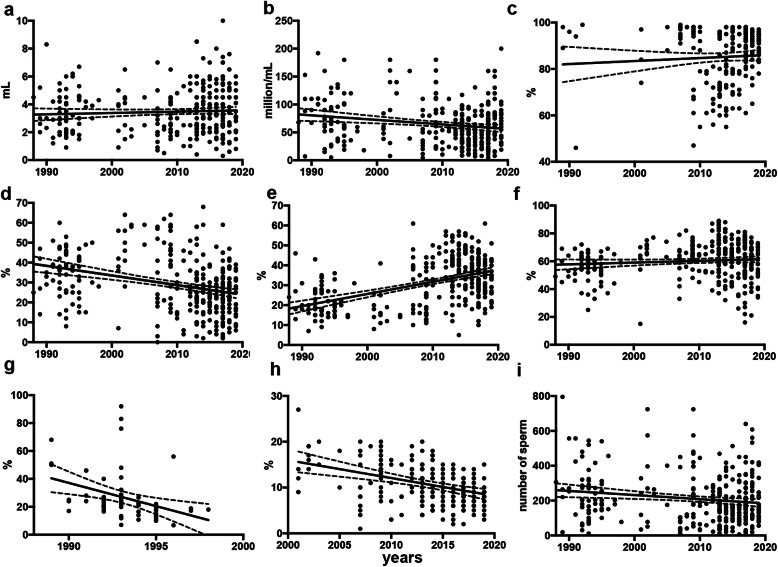
Graphic representation of the relationship between semen parameter values and time. Parameters included are: **a** volume (milliliter) *n* = 317 **b** sperm concentration, (millions of spermatozoa per milliliter of semen), *n* = 317 **c** vitality (percentage of viable sperm over the total) *n* = 209, **d** percentage of fast progressive motility (motility type a), *n* = 317 **e** percentage of slow progressive motility (motility type b), *n* = 317 **f** percentage of total progressive motility (motility types a + b), *n* = 317 **g** percentage of normal sperm morphology from 1988 to 1999 *n* = 65 **h** percentage of normal sperm morphology from 2000 to 2019 *n* = 252 **i** total number of spermatozoa per ejaculate (expressed in millions of cells), *n* = 317

Analysis of the sperm parameter values revealed that there were significant changes over time in sperm concentration, fast sperm motility (grade a), slow sperm motility (grade b), and normal sperm morphology while the other semen characteristics were not modified (Fig. 1 and Table 3).

**Table 3:** Analysis of correlation of sperm parameter values with time

	Volume (mL)	Concentration (10^**6**^xml)	Motility a (%)	Motility b (%)	Vitality (%)	Normal morphology (%)	Progressive motility (a + b)	Total motility (%)
Spearman’s rho	0.067	−0.149	−0.342	0.397	0.057	−0.636	0.018	−0.0545
*P*-value	0.23	0.008	< 0.0001	< 0.0001	0.409	< 0.0001	0.75	0.34

Correlation results between sperm parameters and time were expressed by the Spearman correlation coefficient and its corresponding *p*-value

Sperm concentration was expressed as millions of sperm cells per milliliter, and evaluation of its changes over time was determined by a Spearman correlation analysis. Semen volume did not show variations during the 30 years nor did sperm vitality (Fig. 1a, c). Sperm concentration decreased significantly over the 30 years by 0.9 million/mL per year *R*^2^ = 0.03 (Fig. 1b and Table 3).

The percentage of spermatozoa moving with fast progressive motility (motility a) statistically significantly decreased over time (Fig. 1d). However, the slow progressive motility pattern (motility b) significantly increased (Fig. 1e). There were no statistical differences either in progressive motility (motility a + b) or in total motility (motility a + b + c) during the 30 years (Fig. 1f and Table 3).

**Fig. 2:**
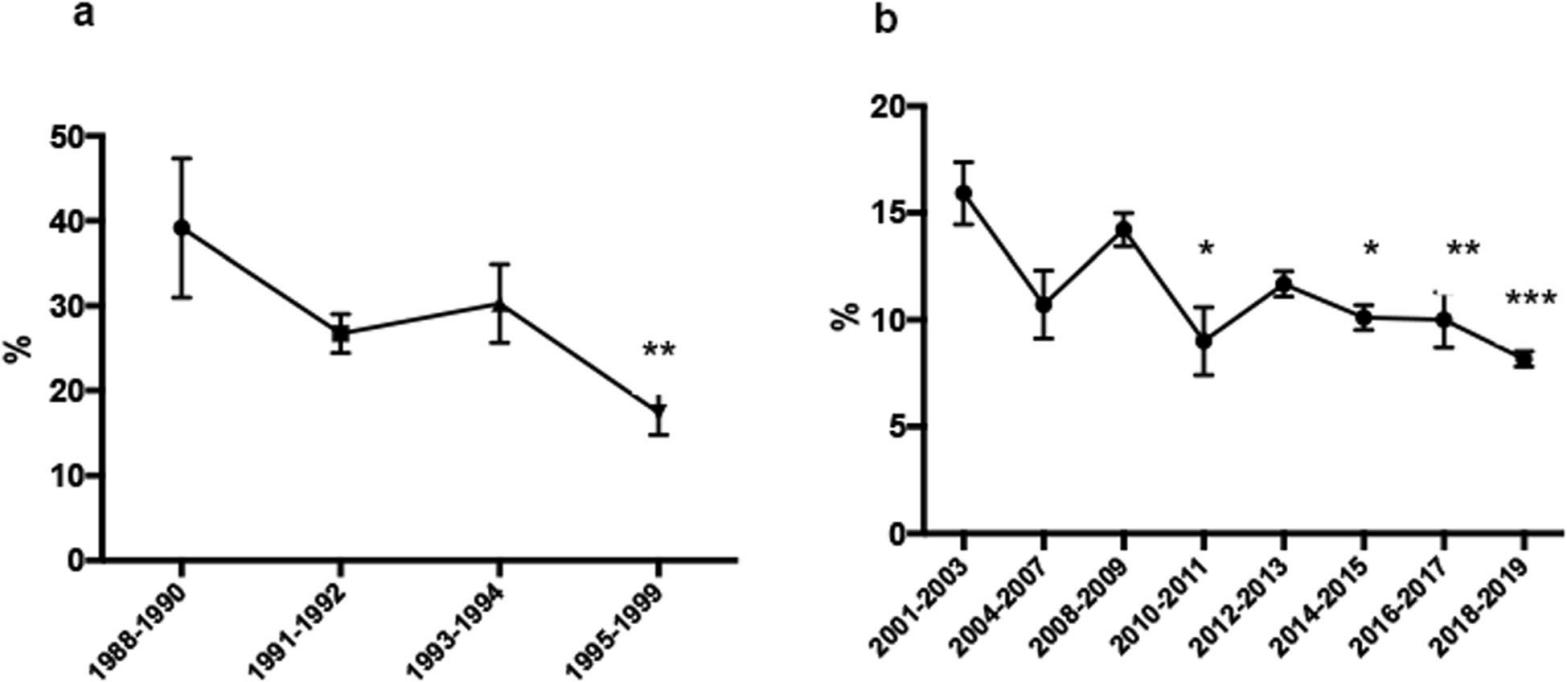
Graphic representation of the relationship between the donor’s sperm normal morphology and time. The mean of sperm considered normal was calculated in periods of 30 months and shown with time. **a** Graphic representation of the variation of the percentage of morphologically normal spermatozoa between the period 1988–1999, *n* = 65. **b** Modifications of normal sperm morphology between 2000 and 2019, *n* = 252. One way ANOVA test, Tukey post-test *p < 0.05, **p < 0.01,***p < 0.001

Sperm morphology may reflect the applied methodology at the moment of the analysis. Consequently, data analysis was performed by dividing the sample into two groups according to the date of men’s recruitment (before or after 2000). In both groups, the correlation analysis demonstrated a decline in the percentage of spermatozoa with normal morphology through time. In the case of data collected before the year 2000: Spearman correlation analysis showed an r coefficient of − 0.5388, *p*-value < 0.0001 *n* = 63 (Fig. 1g). After 2000 when strict Kruger criteria began to be applied, data showed Spearman r coefficient of − 0.4185 *P* < 0.0001 *n* = 250 (Figs. 1h and 2). Data were arbitrarily divided into groups of 3–4 years in both periods to include a similar number of donors in each group. Normal sperm morphology decreased from 39.2 ± 20.2 (mean ± SD) in the period 1988–1990 to 17.4 ± 10.2 in 1997–1999 (Mann-Whitney test, *p* < 0.03). Normal sperm morphology decreased from 15.9 ± 5.0 (mean ± SD) in 2001–2003 to 8.2 ± 2.5 in 2017–19 (Student’s t-test, *p* < 0.05).

We found that the semen volume did not statistically change with time (Fig. 1a and Table 3).

The total sperm number of the analyzed semen samples was not modified during the period of the study (Spearman r − 0.08764, *p* = 0.1194 and linear regression analysis: *R*2 = 0.022) (Fig. 1i).

### Men’s epidemiological characteristics and their relation with semen parameter values with time

To evaluate if the variations of semen parameter values with time were dependent on the epidemiological characteristics of the subjects, the entire population was stratified into three groups, each group representing the total number of men recruited in approximately 10 years (Table 4). We observed differences in smoking consumption with a decrease in the number of smokers.

**Table 4:** Evolution of epidemiological characteristics of fertile men candidate for semen donation over time

	1988–2000	2001–2010	2011–2019
**N**	64	56	197
**Age (years)**	24.4 ± 4.9	23.4 ± 3.4	24.9 ± 4.2
**BMI (%)**	24.2 ± 2.8	23.5 ± 2.2	23.6 ± 2.6
**Smokers (%)**	31.1 ^a^	48.0 ^b^	11.3 ^ab^
**Alcohol consumers (%)**	18.2 ^ab^	56.0^a^	48.6^b^
**Drugs (%)**	ND	ND	24

Values with the same superscript are significantly different (*p*-value < 0.05). Numbers are expressed as mean ± SD Standard deviation or percentage of the total number that answered the questionnaire. Statistical analyses of age and BMI were calculated with the Kruskal-Wallis test and ONE way-ANOVA test respectively. Differences of men who smoke and consume alcohol in each group were compared with chi-squared test

*N* Number of individuals, *ND* No data

There was an increase in men who consumed alcohol (Table 4). However, there was no decrease of the mean ± SD of sperm parameter values in men who consumed alcohol vs. those who did not (sperm concentration: 67.9 ± 43 vs. 64.7 ± 36 million/ml, Mann-Whitney test *p* = 0.6; the percentage of progressive sperm motility 59.9 ± 12.2 vs. 61.6 ± 13.4%, Student’s t-test *p* = 0.3, and the percentage of normal sperm morphology 15.9 ± 14.0 vs. 11.8 ± 5.7, Mann-Whitney test *p* = 0.09).

Multiple regression analysis revealed no significant relationship between sperm parameter values and the tested independent variables. Semen volume could not be predicted by men’s age, the consumption of cigarettes or alcohol (F(3,204) = 1.998 *p* = 0.12 R^2^0.03). Sperm concentration had no statistical association with age, smoking or alcohol consumption (F(3,204) = 1.849 *p* = 0.14 *R*^2^ = 0.026). Neither progressive sperm motility (F(3,204) = 0.44 *p* = 0.735 *R*^2^ = 0.006) nor normal sperm morphology (F(3,106) =0.552 *p* = 0.6 *R*^2^ = 0.016) could be predicted by the same analyzed variables.

Drug consumers have been registered since 2012 so data were available only for the last group (2011–2019) (Table 4) and the variable was not included in the multiple linear analyses.

Drug consumption was not statistically associated with modifications in any of the analyzed sperm parameter values. The mean ± SD of sperm parameter values in men who consumed drugs vs. those who did not were: sperm concentration: 61.5 ± 42.5 vs 58.8 ± 26.4 million/ml (Mann-Whitney test *p* = 0.8); the percentage of progressive sperm motility: 60.9 ± 13.5 vs. 58.7 ± 26.4%, (Student t-test, *p* = 0.8), and the percentage of normal sperm morphology: 9.8 ± 8.0 vs. 9.9 ± 3.7 (Mann-Whitney test *p* = 0.4), respectively.

## Discussion

In this work, we analyzed changes in semen quality in the past 30 years. We chose a population of semen donors who voluntarily responded to the requests that are periodically issued from the Andrology laboratory (Fertilab). The purpose of Fertilab is to recruit men who desire to participate in the sperm donation programs, meaning that the study population is composed of young men (under 36 years old). Moreover, we did not include men carrying pathologies that may compromise their fertility (e.g. diabetes, extreme obesity, cancer, or high blood pressure) meaning that data collected may be considered coming from a group of young healthy men. We found that there was a decrease in the semen parameter values from these possibly fertile men over the years. Specifically, sperm concentration, morphology, and motility type “a” decreased during the analyzed period. No statistically significant evidence was found to support the hypothesis of impairment of other parameters, eg: total progressive motility, sperm vitality, or semen volume.

Previous work has shown that the decrease of sperm concentration may reflect an increase in semen volume, therefore overriding any significant modifications of these parameters [22]. In comparison with the mentioned study, by analyzing 317 donors instead of 71 men, we did not detect an increase in semen volume, so our data support a real decrease in sperm numbers with time.

Sperm concentration and the volume of the ejaculate have been the two parameters most often studied with time. Sperm concentration decreased during that period. Our results are in accordance with similar studies including candidates for sperm donation [13, 27]. The measure of sperm concentration and volume of the ejaculate is considered more objective and precise than sperm motility and morphology [13]. These parameters may not depend on differences between laboratory or technician procedures and are those that are more consistently found as varying with time [6, 13, 27]. On the contrary, motility and morphology have more bias that depends on methods and even different classification criteria. In the case of motility, we observed that the percentage of fast progressive motility (motility a) decreased but it was accompanied by an increase of motility b. Altogether, these results indicate that there were no statistical differences in progressive motility (motility a + b) during the 30 years (Fig. 1f and Table 3).

Consequently, progressive motility did not change during the period analyzed. Progressive motility is considered to be less subjective than classifying sperm motility according to sperm speed so the current analysis is generally informed using a + b motility [25]. We conclude that actual sperm motility was not modified during the time of our study.

Regarding the variation in the morphology of spermatozoa, this study found that the percentage of spermatozoa considered normally formed has also suffered a marked decrease, as established by the correlation analysis applied for this variable. To observe this association, it was necessary to segment the population into two groups: those individuals studied between 1988 and 2000, and those studied from 2000 onwards until 2019. This separation is justified by the different morphological evaluation criteria carried out in the Fertilab andrology laboratory during such periods. In this sense, a change was made to these criteria during the years. In the last 25 years, researchers began to use “strict Kruger criteria” [26]. This not only lowered the cutoff points considered “normal” but also implied have a stricter classification of a spermatozoon as normal. In the case of Fertilab, the change has been introduced since January 2000. The analysis of both groups allowed us to establish a decrease in the percentage of spermatozoa considered normal over time independently of the methodology or the classification criteria.

The decrease in sperm parameters during the last years is multi-causal. Spermatogenesis in man compared with that in other mammals is particularly vulnerable to external factors and humans are more likely to be at greater risk from toxic agents [16, 28]. We analyzed possible environmental factors associated with the decline in sperm parameters and men’s fertility, such as the man’s age and obesity (through BMI) as well as exposure to toxic substances. Our study considered only healthy men between 18 and 36 years old since this is one of the requirements for being accepted as a sperm donor. So it is not unexpected that both the age and the BMI of the individuals were not associated with any change in semen parameter values. A decrease in semen parameter values is expected from the age above 40 years old [29]. Similarly, the mean BMI in the analyzed sample was 23.74 (SD ± 2.56), with most of the individuals corresponding to the “normal weight” category, meaning that in this study the already known association of sperm parameters and a high BMI was not present [30]. Consequently, the observed decrease of semen parameter values occurs in an otherwise healthy population of possible fertile men.

When assessing alcohol, tobacco, and marijuana consumption, this study did not detect a significant association with any of the sperm quality parameters. The number of men that declare to consume alcohol and drugs increased during the period but the consumption of tobacco decreased. Since 2003–2004 policies against tobacco have increased in Uruguay and diminished the number of smokers [31]. However, the use of Cannabis (principal drug in this study) has become more flexible in Uruguay in 2013 and the consumption has been more accepted, probably increasing its use, especially among adults [32]. Therefore, the data collected in this study may mirror the habits of Uruguay’s population during the period. We have not been able to establish the amount of consumption by donors, thus the possible dose-dependent effects could not be determined as it was in other studies [15, 33]. However, our results underline the importance of analyzing the data in the context of geographical and environmental characteristics of the population.

Altogether, our results suggest that, in Uruguay, as in other countries such as the United States, European and Asian countries, there is a trend of sperm quality to decrease [6, 9, 10, 13, 16, 34, 35]. These results are in contrast to that proposed by authors such as Multigner and Oliva [36] and Tortolero [37], who showed that the tendency of semen quality to deteriorate was not present in South American countries such as Argentina, Venezuela, and Brazil. However, it is important to highlight the lack of studies that can be widely extrapolated to the general population of these countries, as well as the existence of few studies that describe the current situation in terms of fertility and seminal quality in this region of the globe.

We acknowledge some limitations of the study, e.g. the number of samples considered is heterogeneously distributed, with a higher concentration of data in the last 10 years. We were not able to recognize what the causes that generated this asymmetric distribution of the sample were, but they could be related to the greater availability and dissemination of information regarding semen banks and greater accessibility to donation programs, among other reasons. Regarding the representativeness of the sample, we must mention that the data were obtained from a single laboratory, considering a well-defined population of individuals, which makes it difficult to extrapolate the results obtained for other populations. The number of men analyzed in this study is lower than others that also analyzed sperm donors [10, 13] but it has the strength that includes a defined population of presumably healthy men. We also highlight the absence of data on occupation, exposure to environmental factors, and ethnic origin, as well as the missing data regarding some donors (e.g missing data on smoking or alcohol consumption). In the future, we intend to correlate these data with a paired sample of individuals who consulted during the same periods for infertility as well as adding information about environmental factors.

Finally, we disbelieve that the decline in the quality of semen over time could be attributed to changes in technical personnel, techniques, or equipment. During the 30 years of the study, there were few staff changes. Fertilab is the only sperm bank in the country and it has been under the same directive committee and strict quality controls. The technicians had the same training, the method of the study did not change, except for morphology, and it was regularly controlled.

## Conclusions

Semen quality in Uruguay has decreased in the last 30 years mainly in the sperm cell number and sperm morphology. These data should be a warning about a possible drop in male fertility. We must bear in mind, however, that the reported mean value at the end of this study was above the values considered normal by the WHO. In the future, it will be necessary to follow up sperm parameters to check if the trend continues and to identify possible causes.

## Data Availability

Data supporting the results reported in this article are available from the corresponding author on reasonable request.

## Abbreviations

BMI: Body mass index
P25: 25th centile
P75: 75th centile
ND: No data
NS: Not significant
SE: Standard error
WHO: World Health Organization

## References

[1] Mascarenhas MN, Flaxman SR, Boerma T, Vanderpoel S, Stevens GA (2012). National, regional, and global trends in infertility prevalence since 1990: a systematic analysis of 277 health surveys. PLoS Med.

[2] Skakkebaek NE, Jørgensen N, Andersson A-M, Juul A, Main KM, Jensen TK (2019). Populations, decreasing fertility, and reproductive health. Lancet.

[3] GBD 2017 Population and Fertility Collaborators (2018). Population and fertility by age and sex for 195 countries and territories, 1950–2017: a systematic analysis for the Global Burden of Disease Study 2017. Lancet.

[4] GBD 2019 Demographics Collaborators (2020). Global age-sex-specific fertility, mortality, healthy life expectancy (HALE), and population estimates in 204 countries and territories, 1950–2019: a comprehensive demographic analysis for the Global Burden of Disease Study 2019. Lancet.

[5] Skakkebaek NE, Rajpert-De Meyts E, Buck Louis GM, Toppari J, Andersson A-M, Eisenberg ML (2016). Male reproductive disorders and fertility trends: influences of environment and genetic susceptibility. Physiol Rev.

[6] Carlsen E, Giwercman A, Keiding N, Skakkebaek NE (1992). Evidence for decreasing quality of semen during past 50 years. BMJ.

[7] Auger J, Kunstmann JM, Czyglik F, Jouannet P (1995). Decline in semen quality among fertile men in Paris during the past 20 years. N Engl J Med.

[8] Irvine DS (1997). 3 declining sperm quality: a review of facts and hypotheses. Baillières Clin Obstet Gynaecol.

[9] Swan SH, Elkin EP, Fenster L (2000). The question of declining sperm density revisited: an analysis of 101 studies published 1934–1996. Environ Health Perspect.

[10] Liu J, Dai Y, Li Y, Yuan E, Wang Q, Wang X (2020). A longitudinal study of semen quality among Chinese sperm donor candidates during the past 11 years. Sci Rep.

[11] Benshushan A, Shoshani O, Paltiel O, Schenker JG, Lewin A (1997). Is there really a decrease in sperm parameters among healthy young men? A survey of sperm donations during 15 years. J Assist Reprod Genet.

[12] Andolz P, Bielsa MA, Vila J (1999). Evolution of semen quality in North-eastern Spain: a study in 22 759 infertile men over a 36 year period. Hum Reprod.

[13] Splingart C, Frapsauce C, Veau S, Barthélémy C, Royère D, Guérif F (2012). Semen variation in a population of fertile donors: evaluation in a French centre over a 34 year period. J Androl.

[14] Paasch U, Grunewald S, Kratzsch J, Glander H-J (2010). Obesity and age affect male fertility potential. Fertil Steril.

[15] Sharma R, Harlev A, Agarwal A, Esteves SC (2016). Cigarette smoking and semen quality: a new meta-analysis examining the effect of the 2010 World Health Organization laboratory methods for the examination of human semen. Eur Urol.

[16] Rahban R, Nef S (2020). Regional difference in semen quality of young men: a review on the implication of environmental and lifestyle factors during fetal life and adulthood. Basic Clin Androl.

[17] Jensen TK, Gottschau M, Madsen JOB, Andersson A-M, Lassen TH, Skakkebæk NE (2014). Habitual alcohol consumption associated with reduced semen quality and changes in reproductive hormones; a cross-sectional study among 1221 young Danish men. BMJ Open.

[18] De Brucker S, Drakopoulos P, Dhooghe E, De Geeter J, Uvin V, Santos-Ribeiro S (2020). The effect of cigarette smoking on the semen parameters of infertile men. Gynecol Endocrinol.

[19] Siqueira S, Ropelle AC, Nascimento JAA, Fazano FAT, Bahamondes LG, Gabiatti JR (2020). Changes in seminal parameters among Brazilian men between 1995 and 2018. Sci Rep.

[20] Sengupta P, Dutta S, Krajewska-Kulak E (2017). The disappearing sperms: analysis of reports published between 1980 and 2015. Am J Mens Health.

[21] Ravanos K, Petousis S, Margioula-Siarkou C, Papatheodorou A, Panagiotidis Y, Prapas N (2018). Declining sperm counts… or rather not? A mini review. Obstet Gynecol Surv.

[22] Barrera N, Ordoqui R, Montes JM, Canepa M, Bonelli C, Surka C (2020). The Uruguayan semen donor population: a twenty-eight-year retrospective study. Andrologia.

[23] World Health Organization (1980). Laboratory manual for the examination of human semen and semen cervical interactions. Distributed by WHO Special Programme of Research Development and Research Training in Human Reproduction.

[24] World Health Organization (1999). WHO laboratory manual for the examination of human semen and sperm-cervical mucus interaction.

[25] World Health Organization (2010). WHO laboratory manual for the examination and processing of human semen.

[26] Kruger TF, Swanson RJ, Hamilton M, Simmons KF, Acosta AA, Matta JF (1988). Abnormal sperm morphology and other semen parameters related to the outcome of the hamster oocyte human sperm penetration assay. Int J Androl.

[27] Gyllenborg J, Skakkebaek N, Nielsen N (1999). Secular and seasonal changes in semen quality among young Danish men: a statistical analysis of semen samples from 1927 donor candidates during 1977-1995. J Androl.

[28] Sharpe RM (2010). Environmental/lifestyle effects on spermatogenesis. Philos Trans R Soc Lond Ser B Biol Sci.

[29] Verón GL, Tissera AD, Bello R, Beltramone F, Estofan G, Molina RI (2018). Impact of age, clinical conditions, and lifestyle on routine semen parameters and sperm kinematics. Fertil Steril.

[30] Agarwal A, Majzoub A, Parekh N, Henkel R (2020). A schematic overview of the current status of male infertility practice. World J Mens Health.

[31] Gravely S, Fong GT, Driezen P, McNally M, Thrasher JF, Thompson ME (2016). The impact of the 2009/2010 enhancement of cigarette health warning labels in Uruguay: longitudinal findings from the International Tobacco Control (ITC) Uruguay survey. Tob Control.

[32] Hall W, Lynskey M (2020). Assessing the public health impacts of legalizing recreational cannabis use: the US experience. World Psychiatry.

[33] Maris E, Huberlant S, Torre A (2017). Tabaco y fertilidad. EMC - Ginecología-Obstetricia.

[34] Mehta J, Woodward B. Male infertility. Sperm Diagnosis, Management and Delivery. New Delhi: JP Medical Ltd; 2014.

[35] Borges E, Setti AS, Braga DP d AF, Figueira R d CS, Iaconelli A (2015). Decline in semen quality among infertile men in Brazil during the past 10 years. Int Braz J Urol.

[36] Multigner L, Oliva A (2002). Secular variations in sperm quality: fact or science fiction?. Cad Saude Publica.

[37] Tortolero I, BellabarbaArata G, Lozano R, Bellabarba C, Cruz I, Osuna JA (1999). Semen Analysis in men from Mérida, Venezuela, Over a 15-year period. Arch Androl.

